# Early-Life Temperamental Differences as Longitudinal Predictors of Unintentional Injuries

**DOI:** 10.1093/jpepsy/jsad072

**Published:** 2023-10-17

**Authors:** Albert J Ksinan, Andrea Dalecká, Lubomír Kukla, Hynek Pikhart, Martin Bobák

**Affiliations:** RECETOX, Faculty of Science, Masaryk University, Czech Republic; RECETOX, Faculty of Science, Masaryk University, Czech Republic; RECETOX, Faculty of Science, Masaryk University, Czech Republic; RECETOX, Faculty of Science, Masaryk University, Czech Republic; Department of Epidemiology & Public Health, University College London, Institute of Epidemiology and Health Care, UK; RECETOX, Faculty of Science, Masaryk University, Czech Republic; Department of Epidemiology & Public Health, University College London, Institute of Epidemiology and Health Care, UK

**Keywords:** injury, latent profile analysis, longitudinal, personality, prevention, temperament

## Abstract

**Objective:**

Unintentional injuries are the leading cause of hospitalization and death among children. Compared to environmental factors, less attention in injury preventive efforts has been paid to how individual characteristics relate to the risk of injury. Using a large prospective cohort, the current study assessed the longitudinal impact of early-life temperament on the cumulative number of injuries until mid-adolescence.

**Methods:**

The data came from the European Longitudinal Study of Pregnancy and Childhood (ELSPAC-CZ). Temperament was evaluated by mothers when children were 3 years old (*N* = 3,545). The main outcome was the pediatrician-reported sum of child’s injuries from age 3 to 15 (seven timepoints). Latent profile analysis (LPA) was used to determine classes based on temperamental dimensions and then extended to a mixture model with a distal count outcome. The covariates included maternal conflict and attachment, sex, family structure, and maternal education.

**Results:**

The LPA determined the existence of three classes: shy children (8.1% of the sample; lowest activity/highest shyness), outgoing children (50.8%; highest activity/lowest shyness), and average: children (41.1%; middle values). Results from a mixture model showed that the outgoing temperament was associated with the highest longitudinal risk for injuries, as both average children (IRR = 0.89 [0.80, 0.99]), and the shy children (IRR = 0.80 [0.68, 0.95]) had lower risk.

**Conclusions:**

Early childhood temperamental differences can have long-term effects on injury risk. Highly active children showed the highest risk for future injuries, suggesting that these characteristics make them more likely to be involved in novel and potentially dangerous situations.

## Introduction

Unintentional injuries including falls, road traffic injuries, poisoning, drowning, or fire-related burns are the leading cause of hospitalization and death among children and adolescents. Global progress has been achieved in reducing childhood mortality in the last 30 years, as the total number of deaths of children under five decreased worldwide from 12.8 million in 1990 to 5 million in 2021 (World Health Organization, 2021). However, substantial regional differences have been preserved, both globally and among European countries, highlighting significant inequalities between low/middle- and high-income countries (World Health Organization, 2008).

Compared to other European countries, the Czech Republic belongs among those regions with a higher proportion of under-5 deaths caused by unintentional injuries. Based on the latest data from 2017, the Czech Republic ranks 36th out of 53 European countries with a 0.07% death rate, a rate higher than Ireland, Denmark, Finland, Norway, Croatia, Italy, Slovakia, or the United Kingdom (World Health Organization, 2020). Moreover, the results from the European report on child injury prevention published by World Health Organization showed that hospitalization rates for children and adolescents were approximately twice as high in the Czech Republic, compared to Netherlands or Portugal.

Through the implementation of various preventative measures and the regulation of environmental factors, injury-related deaths among children aged 5 to 14 decreased by 50% between 1990 and 2018, according to the World Health Organization (2019). Restricting access to hazardous chemicals; adding safety locks on windows, doors, and stairways; or making cars and playgrounds safer are effective tools that substantially reduce the occurrence as well as the severity of injuries (Tupetz et al., 2020). Beyond simply preventing access to dangerous agents, parents and their attitude toward the risk of injury play a significant role in child injury prevention. Several previous studies showed that parents perceived injuries as an inevitable part of childhood rather than an event that might be prevented (Whitehead & Owens, 2012). Previous research also emphasized the importance of parenting practices in the risk of childhood injury. Chiefly among them, parental supervision has been found to be negatively associated with a risk for injury (Morrongiello et al., 2006; Schnitzer et al., 2015). Notably, previous research revealed that parental supervision can interact with child temperament to prevent risk of injures, typically among impulsive and sensation-seeking children (Morrongiello & McArthur, 2018; Schwebel et al., 2004). In addition, permissive parenting has been associated with an increased likelihood of injury (Bijttebier et al., 2003; Plumert & Schwebel, 1997; Schwebel & Gaines, 2007). Moreover, the parent–child relationship has been previously reported as an important predictor of childhood injuries (Schwebel et al., 2011). For instance, a father’s positive relationship with children had a protective influence on childhood injuries (Schwebel & Brezausek, 2010).

Previous research has also identified several individual characteristics of children that are associated with the risk of getting injured—this has been historically referred to as an injury or accident proneness (Visser et al., 2007). The history of research on individual risk for injuries has been mired in controversy as it was often perceived as blaming individuals for their mishappenings (Bijttebier et al., 2003; Grossman, 2000). Nevertheless, the risk for injuries is non-random, as it varies in relation to individual behavioral characteristics. Chiefly among them is a child’s temperament which affects how they approach novel situations and how they assess risks (Vollrath et al., 2003). Temperament is conceptualized as the normal variation of characterological differences in emotional response and intensity (Clauss et al., 2015).

Various conceptualizations of temperament have been put forth, with one influential example being the EAS temperament model proposed by Buss and Plomin (1984). This model posits the existence of three fundamental temperament dimensions, which are heritable, developmentally stable, and noticeable in early childhood: emotionality (defined as distress accompanied by intense automatic arousal; Buss, 1991), activity (individual differences in expenditure of physical energy; Buss, 1991), and sociability/shyness (motivation to seek other people vs. inhibited, anxious, or self-conscious behavior; Buss, 1991). These dimensions can be effectively mapped onto other important temperament/personality frameworks, such as the Big Five model, where emotionality resembles neuroticism, activity resembles extraversion, and sociability/shyness resembles agreeableness (Walker et al., 2017).

Many studies have explored the associations between temperamental dimensions and injuries. The most important temperamental characteristic involves active, impulsive, or undercontrolled behavior patterns, which have been repeatedly found to lead to a higher risk of injuries among children (Karazsia & van Dulmen, 2008; Rowe et al., 2007; Schwebel & Plumert, 1999). Conversely, suppressing inappropriate responses during new and uncertain situations is recognized as good inhibitory control and has been found to be a protective factor for risk of injury in several longitudinal studies (Schwebel, 2004; Schwebel & Plumert, 1999; Wu et al., 2023). The impulsivity dimension is reflected in activity and partially sociability/low shyness dimensions of EAS. Similarly, shyness in childhood has been shown as a protective factor from getting injured (Myhre et al., 2012). Objective observations in playgrounds suggested that shy children tend to avoid new social interactions with their peers that might protect them from potentially risky situations (Tang et al., 2022).

Nevertheless, important questions remain unanswered based on the existing research on temperamental correlates of injury risk. The few existing longitudinal studies have mostly covered shorter time periods (such as several years) and given the relative scarcity of injuries in one’s life, the effect of temperament might become pronounced within a longer period. Furthermore, the existing studies on temperament have focused on levels of temperamental dimensions (such as impulse control or activity), that is, a variable-centered approach. Using a person-centered approach would provide further advancement beyond existing variable-based methods. The strength of the person-centered approach lies in its ability to reveal complex interplays among multiple variables within heterogeneous subgroups. By considering the unique temperamental profiles of children, this approach enables us to discover specific subpopulations within the sample (defined as combinations of levels of temperamental dimensions) that would be otherwise overlooked by a conventional variable-oriented approach, which focuses on averaged parameters drawn from a single population (Bergman & Magnusson, 1997; Howard & Hoffman, 2018).

Beyond temperamental differences, children’s demographic characteristics also play an important role in injury risk. Multiple studies have found that boys are more likely to get injured than girls, and their injuries are generally more severe (Bijttebier et al., 2003; Matheny, 1991; Morrongiello & Rennie, 1998). There are several reasons why this might be the case. One reason might be that boys are biologically prone to higher levels of impulsivity and sensation seeking (Cross et al., 2011), which is reflected in engaging in more dangerous activities and a more careless approach and assessment of an individual’s skill (Alonso-Fernández et al., 2017). Furthermore, the types of activities in which boys and girls find themselves differ. Boys tend to spend more time outdoors, unsupervised, and engage in more physically demanding activities, whereas girls are more likely to be involved in more indoor and less dangerous activities (Rosen & Peterson, 1990; Wang et al., 2018). Finally, how boys and girls are socialized might explain these differences too. Boys are encouraged to take more risks (Morrongiello & Dawber, 2000) and the parental reaction to their (non-life-threatening) injuries is often more cavalier than when girls suffer the same injuries (Bijttebier et al., 2003). Furthermore, the type and the context of children’s injuries vary with age. For instance, poisoning, burns, and drownings are most prevalent in preschool-aged children, while falls most commonly occur among school-aged children (Agran et al., 2003; Peng et al., 2018). Not surprisingly, injuries of toddlers and infants mostly appear at home, whereas injuries to school-aged children tend to occur outside of their homes (Bijttebier et al., 2003).

Moving from individual to structural effects, several studies have focused on the impact of social inequalities, suggesting that children growing up in lower-income households are more likely to experience unintentional injuries (Campbell et al., 2019; Ma et al., 2010). Several underlying mechanisms have been proposed to explain this association, including the role of the child’s environment and child supervision. Living in less advantaged households with a lack of child safety equipment and a greater chance of playing on the street rather than in safe playgrounds contributes to a greater risk of unintentional injury during childhood. Moreover, lower health literacy together with various stressors of parents with lower socioeconomic status might lead to impairment of supervision and thus increase the risk of injury (Campbell et al., 2019). The family type has an additional impact on a child’s injury risk as single parents may have less time and resources for child-rearing (O’Connor et al., 2000).

Most of the existing studies on injury risk among children have been limited by relying on small samples, a short time span, or retrospective reports of injuries. Previous studies on childhood injuries have been conducted mostly in the United States and Western Europe. The Central and Eastern European countries have undergone substantial socioeconomic and political transformations over the past three decades, but a study on longitudinal factors associated with childhood injuries in this unique context has been lacking (Bobak et al., 2000). The current study seeks to fill these gaps by assessing the longitudinal effect of temperament, measured early in life, on the cumulative number of pediatrician-reported injuries until mid-adolescence, using a large Central European prospective cohort. Specifically, we expect that children with high levels of activity will be at higher risk for unintentional injuries. On the other hand, we expect that children with high levels of shyness will be at a lower risk for injuries. Given the lack of evidence for the association between emotionality and unintentional injuries, we propose no specific hypothesis regarding the effect of this dimension.

## Methods

### Sample

The sample for this study came from the Czech part of the European Longitudinal Study of Pregnancy and Childhood (ELSPAC). This is a prospective birth cohort that started in 1990, initially enrolling pregnant women from Brno and Znojmo towns in the Czech Republic. Information from 5,151 mothers and their newborns was available at the baseline (Piler et al., 2017). Data collection included self-reported questionnaires completed by both parents, pediatricians, and later by teachers and children themselves. For this study, we employed mothers’ and pediatricians’ reports, as fathers only provided self-reports. Throughout the study, the pediatricians and mothers reported on children when they were 6 months old, 18 months, and then when the children were 3, 5, 7, 11, 15, 18, and 19 years old. For pediatrician reports of injuries, we used reports from 3 to 15 years due to substantial data attrition after age 15, for a total of five timepoints, spanning 12 years. For mother reports, we used reports from when the children were 3 years old. The analytic sample at age 3 was 3,639. After removing problematic cases (see Plan of Analysis), the final analytic sample was *N* = 3,534. Informed consent was obtained from all participants in the study during each wave of data collection. The data underlying this article were provided by the Central European Longitudinal Study of Pregnancy and Childhood (CELSPAC) research infrastructure (ELSPAC data administrator). . Access to ELSPAC data is available to researchers upon request.

### Measures

#### Main Outcome

**Injuries.** In the Czech context, every child must undergo a pediatrician appointment once every 2 years. At each timepoint of the study, children’s pediatricians provided the history of injuries since the previous timepoint by reporting the injury/injuries from the child’s medical records using the ICD-9 nomenclature. Before age 11, the questionnaires for pediatricians only provided space for reporting up to three injuries maximum. Starting from the age of 11, the relevant items changed so that pediatricians could also report the total number of injuries suffered by the child since the last timepoint as a separate item, in addition to the items listing the specific injuries and the dates. We used the higher number for items where the total number of injuries reported was higher than those listed. The outcome variable is a cumulative sum of injuries suffered from 3 to 15 years of age.

#### Main Predictor

**Temperament.** Temperamental dimensions were measured by the EAS Temperament Survey for Children: Parental Ratings (Buss & Plomin, 1984), reported by mothers when their children were about three years old. This 20-item questionnaire was originally developed to measure three dimensions: emotionality (tendency to become aroused easily and intensely), activity (levels of activity and speed of action), shyness (tendency to be inhibited and awkward in new situations), with sociability (tendency to prefer the presence of others to being alone) added later. Our preliminary psychometric validation showed that the sociability factor was strongly negatively correlated with shyness (*r *=* *−.71), indicating that they were are measuring a very similar construct, a finding also reported by other researchers (Boer & Westenberg, 1994; Stringaris et al., 2010), possibly related to the age of target children (the two dimensions being undistinguishable among infants or toddlers; Boer & Westenberg, 1994). For this reason, we decided to omit the dimension of sociability. This three-factor scale (5 items per factor) was tested within a confirmatory factor analysis (CFA) framework to provide psychometric validation.

#### Covariates

**Sex of the Child.** Sex of the child was reported as a dichotomous variable, with male as the reference group.

**Family Structure.** The family structure was reported when children were approximately three years old. We dichotomized this variable into two categories: two-parent families (reference group) and single-parent families.

**Maternal Education.** The highest attained education of mother was assessed at study baseline (during mother’s pregnancy). This was recoded into three categories: less than high school education (elementary school or trade school; reference group), high school education/some college (without degree), and college degree (undergraduate or graduate).

**Maternal Attachment.** The maternal attachment was assessed using the mean of three questions, which reflect the mother-rated behavior of children when reunited with their mothers after a period of absence. The items are “My child avoids me when we are reunited,” “My child pushes me away when we are reunited,” and “My child wants a hug when we are reunited” (reverse coded). All three were rated using the following options: *always*, *sometimes*, *hardly ever*, with higher scores indicating secure attachment style (Bowen, 2015, 2017).

**Conflict With Child.** Conflict with child was assessed by asking mothers a question about “How often they have battle of wills with their child,” rated with options *never*, *hardly ever*, *sometimes*, *always* (Culpin et al., 2020).

### Plan of Analysis

First, we checked the responses on the EAS scale to see whether response patterns (responding carelessly) would be present using the *responsePatterns* R package (Gottfried et al., 2022). This led to the removal of *n *=* *105 problematic cases. Then, the descriptive statistics of study variables were computed. After this, we fit the EAS scale in CFA and removed items with low loadings or high factor crossloadings, resulting in an 11-item modified version. Then, we exported the three-factor scores. These extracted factor scores were used for latent profile analysis (LPA). Latent profile analysis is a person-centered method that focuses on finding latent subgroups in a population based on a set of variables (Howard & Hoffman, 2018; Spurk et al., 2020). In this case, individuals are classified into these subgroups based on distinct combinations of values on EAS dimensions. We ran several models by varying the number of classes. After selecting the best model based on several criteria (AIC, BIC, Entropy, proportion of the smallest class), the resulting class membership was used as a predictor of the cumulative injury in a mixture model with the distal outcome and with other covariates included. As including covariates in assessing the LPA model might affect the class membership and lead to miss-specified models (Nylund-Gibson & Masyn, 2016), stepwise methods for assessing the effect of latent class membership on distal outcomes were developed. From those, the three-step BCH method was found to be robust and reliable (see Nylund-Gibson et al., 2019 for more details on the method). The class membership in the BCH defined as inverse logits reflecting the measurement error of the latent class variable (as opposed to creating categorical variables reflecting modal class assignment), is then used as weights in the third step (the model with the distal outcome). In this way, the classification error for each individual is preserved and used in the analyses, making the results more robust.

**Figure 1.**
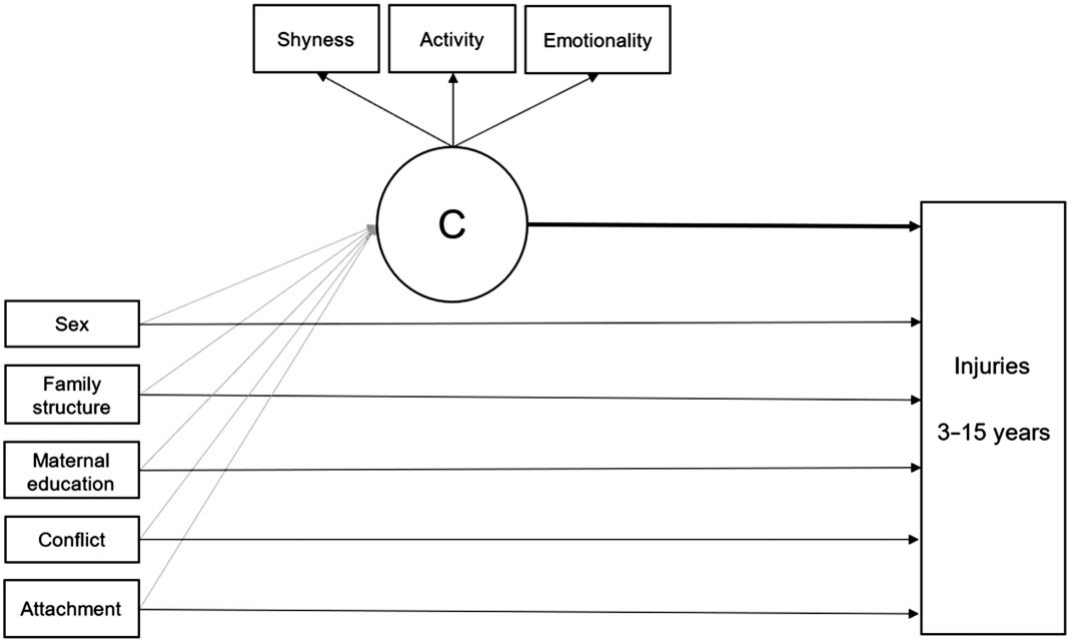
The latent profile analysis with covariates and distal outcome. *Note*. C = class membership; conflict = maternal conflict; attachment = maternal attachment.

In this analysis, the distal outcome (cumulative number of injuries) was modeled as a count variable with a negative binomial distribution. The distal outcome and the class membership were regressed on all the covariates, that is, sex, family structure, maternal education, mother’s attachment, and conflict (see Figure 1 for a graphic representation of the model). Multiple imputation with chained equations method with 50 samples was used to deal with missing data using the R package mice (Buuren & Groothuis-Oudshoorn, 2011) in conjunction with the *countimp* package for imputing count data (Kleinke & Reinecke, 2019). The rate of missingness ranged from .03% to 25% based on the variable. The CFA was estimated in R package *lavaan* 0.6.3 (Rosseel, 2012), while the three-step BCH analysis was computed in *Mplus* 8.6 (Muthén & Muthén, 1998–2017).

## Results

The descriptive statistics of study covariates are presented in Table I. The sample consisted of 51.9% boys and 48.1% girls. There were 92.7% of families with both adults in the same household and 7.3% of other family structures. A total of 33.3% of mothers had less than high school as their highest attained education, 45.8% finished high school, and 20.9% received a college degree. Mean maternal attachment was 2.89 (scores ranging from 1 to 3), and the mean of conflict with child was 2.96 (scores ranging from 1 to 4).

**Table I.** Descriptive Statistics of Study Covariates

	*M/%*	*SD*
Sex		0.50
Male	51.87%	
Female	48.13%	
Family structure		0.26
Single-parent family	7.33%	
Two-parent family	92.67%	
Maternal education		0.73
Less than high school	33.33%	
High school/some college	45.75%	
College degree	20.92%	
Maternal attachment	2.89	0.63
Conflict with the child	2.96	0.78

Table II provides descriptives of injuries across the timepoints used in the study. Although the highest absolute number of injuries was between the age 7 and 11 (timepoint 3), this timepoint spanned 4 years. Dividing by the number of years to compute an average number of injuries per year, the highest number of recorded injuries were between the age 11 and 13 (timepoint 4), with an average of 0.12 injuries per year. The mean number of injuries for the whole time span of 12 years was 1.22 injuries. The five most common types of reported injuries were contusion of face, scalp, and neck (6.2% of all injuries), ankle sprain (4.8%), fracture of radius and ulna (4.7%), contusion of shoulder and upper arm (4.4%), and concussion with no loss of consciousness (4.4%).

**Table II.** Rates of Injuries per Timepoint

Timepoint		*n* children	Mean injuries	*SD*	Mean injuries per year	Cumulative injuries mean
1	Injuries from 3 to 5 years	3,242	0.20	0.46	0.10	0.20
2	Injuries from 5 to 7 years	3,166	0.17	0.41	0.09	0.37
3	Injuries from 7 to 11 years	2,785	0.37	0.69	0.09	0.74
4	Injuries from 11 to 13 years	2,423	0.25	0.54	0.12	1.00
5	Injuries from 13 to 15 years	2,463	0.23	0.54	0.11	1.22
	Total injuries from 3 to 15 years	2,270	1.22	1.44	0.10	

In the next step, we fit the modified EAS scale in a CFA framework and extracted factor scores for each factor. Our results showed that the model fit was inadequate and subsequent changes were done, resulting in a shorter, 10-item version with adequate model fit (*χ*^2^ (32) = 367.850, *p* < .001, CFI = .958, RMSEA = .059, 90% RMSEA CI [.053, .064]), and reliability: shyness *ω* = .79, emotionality *ω* = .74, activity *ω* = .69 (Supplementary Tables 1 and 2 provide more details on the CFA validation).

Then, these factor scores were used for LPA where we tested models with a variety of classes, from 1 to 5 classes. Table III presents the overview of the models. A 3-class model showed improvement in all metrics as compared to the 2-class model. Compared to the model with 4 classes, the extra class in the 5-class model was too small (<1% of the sample size), making the 5-class solution unstable and too idiosyncratic to the data. The final decision was then between the 3-class and the 4-class models. Looking closer at the identified classes, the 4 classes in the 4-class model reflected 2 of the same classes as in the 3-class model (high on shyness/low on activity, low on shyness/high on activity), while the class with middle values from the 3-class model was split into two mid classes in the 4-class model. Moreover, the relative size of the smallest class in the 4-class model was less than 5% (Nylund et al., 2007). For these reasons, we decided to opt for a more parsimonious and interpretable 3-class model as our final model for further analyses.

**Table III.** Fit Indices for Different LPA Models Tested

Models	AIC	BIC	SABIC	Smallest class count (*n*)	Smallest class size (%)	Entropy
1 Class	15,039.74	15,076.76	15,057.69	3,534	100%	–
2 Class	13,584.60	13,646.30	13,614.53	1,011	28.60%	0.719
**3 Class**	**13,008.52**	**13,094.90**	**13,050.42**	**286**	**8.09%**	**0.761**
4 Class	12,624.73	12,735.80	12,678.60	119	3.37%	0.845
5 Class	12,386.6	12,522.35	12,452.44	30	0.85%	0.880

*Note*. The model in bold was the selected model.

The classes identified by the model differed based on levels of activity and shyness, with mean levels of emotionality not significantly different among groups. Class 1, representing 50.8% of values, showed the highest levels of activity from all three groups and the lowest levels of shyness. Class 2, representing 41.1%, showed values that were in the middle of Class 1 and Class 3. Finally, Class 3, representing 8.1% of values, was the opposite of Class 1—the highest levels of shyness with the lowest levels of activity among all classes. For the next analyses, we will refer to Class 1 as “outgoing children,” Class 2 “average children,” and Class 3 “shy children.”

In the next step, we saved the inverse logits of the class membership as weights to be able to replicate the solution from LPA without covariates in the structural model where covariates and the distal outcome are included. First, we compared the temperamental groups based on the levels of covariates. From these, outgoing children had a much lower likelihood of mother having college degree as compared to both average and shy children and were more likely to live in single-parent household as compared to average children (but not to shy children). On the other hand, outgoing children had higher average reported parental warmth when compared to average children (full results for the inter-group comparisons can be found in Supplementary Table 3). Then, the cumulative rate of injuries was regressed on the latent groups and all the covariates. The results from the structural model with imputed data are presented in Table IV. They showed that girls were less likely to suffer injuries as opposed to boys (IRR = 0.77 [0.71, 0.83]). No significant association was found for family structure (IRR = 1.09 [0.94, 1.27]). Maternal education emerged as a protective factor for the risk of injury, as completed high school education was associated with a lower risk for injury compared to less than high school (IRR = 0.89 [0.81,0.99]), and even lowered risk was found for college education (IRR = 0.83 [0.74, 0.94]). Neither maternal attachment nor maternal conflict was longitudinally associated with the risk of injury.

**Table IV.** Results from Structural Model

	IRR	LL	UL	*p* value
Sex				
Male	1 (ref)			
Female	0.77	0.71	0.83	<.001
Family structure				
Two-parent family	1 (ref)			
Single-parent family	1.09	0.94	1.27	.248
Education				
Less than high school	1 (ref)			
High school/some college	0.89	0.81	0.99	.023
College degree	0.83	0.74	0.94	.004
Maternal conflict	1.02	0.97	1.08	.388
Maternal attachment	1.02	0.95	1.09	.630
Temperament				
Outgoing children	1 (ref)			
Shy children	0.80	0.68	0.95	.011
Average children	0.89	0.80	0.99	.037

*Note.* IRR = Incidence rate ratio; LL = lower limit of 95% confidence interval; UL = upper limit of 95% confidence interval.

Regarding the longitudinal impact of toddlerhood temperamental class on the risk of childhood injuries, the average children (IRR = 0.89 [0.80, 0.99]) and shy children (IRR = 0.80 [0.68, 0.95]) had a significantly lower risk for injury when compared to outgoing children, above and beyond other covariates. This means that outgoing children had the highest risk of injury from all three identified classes of children based on EAS. Shy children and average children did not statistically differ from each other.

## Discussion

The present results show that temperamental differences, even when measured very early in a child’s life, can have long-term impact on their injury risk. Children who were rated as highly active by their mothers when they were 3 years old showed the highest risk of experiencing injuries throughout childhood, suggesting that these characteristics make them more likely to be involved in novel and potentially dangerous situations.

Expectedly, the current findings confirmed the result from many previous studies, which found that boys were more likely to get injured (Bijttebier et al., 2003; Morrongiello & Rennie, 1998). In this study, the male sex was the strongest predictor of injury risk from all tested covariates, showing that boys have a higher risk of injury than girls throughout childhood. What this study adds is that a higher risk of injury for boys was found regardless of temperamental differences, suggesting that temperament itself does not explain away these sex differences. These findings may be explained by the various types of activities performed by boys and girls and the difference in risk-taking strategies among boys and girls (Rosen & Peterson, 1990; Wang et al., 2018). Furthermore, a previous study found that parents communicate with sons and daughters differently even though they engage in the same activities. These various parental responses may subsequently promote higher risk taking by boys and higher perceived injury vulnerability by girls (Morrongiello et al., 2016; Morrongiello & Dawber, 1999). For instance, mothers were found to express greater concerns about injury risks to their daughters compared to their sons (Morrongiello et al., 2010). Mothers also exhibited a higher level of encouragement for risk-taking behaviors in their sons than their daughters (Morrongiello & Dawber, 2000).

Maternal education emerged as a protective factor, showing that the risk of injury decreased by 4% with each additional year of education completed. This confirms findings from several previous studies (Campbell et al., 2019; Ma et al., 2010). The explanation for the association between maternal education and the risk of injuries might be that more educated mothers might have higher levels of health literacy, which includes being more closely involved in monitoring their children’s activities and better at assessing the potential environmental dangers (Campbell et al., 2019; O’Connor et al., 2000). Relatedly, higher maternal education might be a proxy for higher income, and previous research showed that children growing up in lower-income households were more likely to experience unintentional injuries due to playing in more hazardous environments (Campbell et al., 2019). Maternal education was also found to be associated with the temperamental groups, as there was a significantly lower proportion of mothers with college degree among mothers of outgoing children compared to other groups, suggesting that the outgoing children share some traits with their mothers that might be associated unfavorably with attaining higher levels of education, such as impulsivity.

No significant association between maternal attachment or maternal conflict on the risk of injury was observed, showing that these two indicators of the mother–child relationship were not affecting the future risk of injury in our study, confirming the findings from some previous studies (Paquette et al., 2023). Because we used baseline measures of these two variables (when children were 3 years old), our results suggest that their potential role in the hypothesized associations might emerge later in a child’s development.

The main findings of this study indicate that differences in temperament, as observed by mothers when the children were around three years old, had long-lasting impacts on the risk of injury. It confirmed the findings from previous studies, which found that higher levels of impulsivity and activity were associated with a higher risk for injuries (Rowe et al., 2007; Schwebel, 2004; Schwebel & Plumert, 1999). The novel findings in this study relate to defining temperament through underlying latent classes and how they differ in relation to injury. These findings show that (1) the number of injuries during childhood is affected by stable individual characteristics; (2) children differ in these characteristics early in life. The outgoing children were characterized by the highest levels of activity and lowest levels of shyness from all classes. Highly active children direct their energy outwards by engaging in many physically demanding activities, which increases their chance of suffering injuries (Wang et al., 2011). Similarly, low shyness (or high sociability) is related to a high “approach” orientation (Poole & Schmidt, 2022), which makes them more likely to engage in novel and unknown situations, which are also associated with a higher risk for injury (Rowe et al., 2007). Inversely, the shy children in our study were found to show the lowest risk of injuries (although not significantly different from the average group), suggesting that the combination of low activity and high shyness reflects an “avoidance” orientation with more timid and withdrawn responses to new situations and fewer friends (Kagan et al., 1984), which becomes protective against the risk for injuries (Atkins & Matsuba, 2008).

Our research found that early childhood temperamental differences predicted the cumulative number of injuries up until age 15, suggesting that a certain part of injury risk might be genetically-influenced, with temperament as an intermediate phenotype (Ordoñana et al., 2008). There have been relatively few studies examining the heritability of injuries. Although some studies did find genetic effects (Phillips & Matheny, 1995; Rowe et al., 2007), other ones found the genetic effects to be small (Ordoñana et al., 2008) or varying based on age (Salminen et al., 2018). The null findings of the latter two studies might be related to the varying time frames, with the Ordoñana et al. focusing on injuries from birth to age 5, while Salminen et al. found no heritability of injuries when the twins were in their early twenties (but about 25% for middle-aged twins). In this way, it is still unclear whether there is a genetic basis for injuries suffered throughout childhood. Of course, no “gene for injury” exists, and these hypothesized genetic effects might only unravel in specific environments. Conversely, other settings could diminish or even negate these influences, highlighting the complex interplay between individual predisposition and environmental factors in shaping the risk of injuries.

Identifying children at high risk of unintentional injuries based on temperament allows us to tailor public health interventions more precisely. They would concentrate on fostering self-regulation skills and skill-based training methods to increase safety on specific occasions (e.g., safe pedestrian behaviors), particularly in children with high levels of impulsivity (Barton et al., 2007). Additionally, educating caregivers on strategies for managing a child’s emotions and behaviors may substantially reduce the risk of injury (Schwebel, 2019). Finally, increased supervision of impulsive children by caregivers may reduce childhood injury prevalence, as described in previous studies (Morrongiello et al., 2011; Morrongiello & Schell, 2010).

### Strengths and Limitations

The current study has several strengths worth mentioning. First, using a large prospective cohort, we were able to model injuries throughout childhood into mid-adolescence, with the largest sample on this topic to date. Importantly, our reports of injuries were prospective, not relying on retrospective maternal or children’s reports, greatly increasing the validity of the outcome. Using a very early measure of temperament to predict the cumulative risk of injuries throughout childhood, we were partially able to lay out an argument against reverse causality in which children are rated as more active as a result of them getting injured more often. This argument is further bolstered by the use of varying reporters for our variables of interest: the temperament was rated by mothers, whereas the injuries were reported by children’s pediatricians. The use of pediatrician’s records greatly improved the study’s external validity, as it removed several sources of bias that are often present when mother reports are used (recency bias, shared method variance). Furthermore, the rigorous analytical approach used in this study, using the BCH method for modeling the effect of latent classes derived from LPA in conjunction with specific imputation of count outcome, strengthens the psychometric validity of the present findings.

The current study also has several limitations. First, we did not model the severity of the injuries. Although the injuries were reported using the ICD-9 codes, and many codes are by definition more severe than others (e.g., an open fracture vs. bruising), other codes might be more ambiguous, and it was impossible to determine the severity of the injury without the full clinical description.

Moreover, the measures related to parenting available to us were limited in scope (e.g., we could not assess parental supervision or parental responses to a child’s risk-taking strategies). Previous studies suggested a potential interactive mechanism between temperament and parental supervision. The study showed that mothers who were more conscious and had more impulsive or sensation-seeking children showed higher levels of supervision (Morrongiello & McArthur, 2018). Future studies in this area would benefit from a more complex assessment of parental behaviors with regard to their children as it relates to injury risk.

Additionally, the psychometric validity of several variables related to child–parent relationship was limited (e.g., maternal conflict measured by a single item; secure maternal attachment measured by three items). Previous studies also showed that children growing up in more hazardous environments were more prone to experience unintentional injuries (Campbell et al., 2019), yet such information on environmental factors (physical or cultural) was not available in the dataset.

Finally, the analytic timeframe of this study covered the period from childhood to mid-adolescence (age 15) due to substantial data attrition at later timepoints. For this reason, it is unclear whether the associations between temperament and injury risk would continue into adulthood.

## Conclusion

Our findings provide insight into the effect of children’s temperament on risk for injuries. Highly active children showed the highest risk for future injuries, suggesting that these characteristics make them more likely to be involved in novel and potentially dangerous situations. However, further research should be conducted to better understand the underlying mechanisms of the relationship between temperament and unintentional injuries to promote the application of potential temperament-specific interventions. Future research may focus on studying the interactions between individual temperament and environmental factors that may further pronounce as well as attenuate the risk of childhood unintentional injuries.

## Supplementary Material

jsad072_Supplementary_Data
