# Supplementary material for: Early-Life Temperamental Differences as Longitudinal Predictors of Unintentional Injuries
Source: J Pediatr Psychol. 2023 Oct 17;49(1):35–44. doi: 10.1093/jpepsy/jsad072 (PMC10799721; doi:10.1093/jpepsy/jsad072)
Supplement: jsad072_Supplementary_Data [file jsad072_supplementary_data.docx]

Supporting Information

Shortened version of the EAS Scale

The fit of the original 3-factor EAS scale was χ2 (87) = 1920.602, *p* <.001, CFI = .858, RMSEA = .084, 90% RMSEA CI [.081, .087]. Table A1 shows the standardized item loadings:

| Table S1 |  |  |
| --- | --- | --- |
|  |  |  |
| *Item Loadings in the 15-item EAS scale* | | |
|  |  |  |
| Factor | Item | Loading |
| Shyness | 1 | .602 |
|  | 8r | .807 |
|  | **12r** | **.773** |
|  | 14 | .742 |
|  | 20r | .593 |
| Emotionality |  |  |
|  | **2** | **.450** |
|  | **6** | **.179** |
|  | 11 | .640 |
|  | 15 | .782 |
|  | 19 | .664 |
| Activity |  |  |
|  | 4 | .649 |
|  | **7r** | **.474** |
|  | 9 | .495 |
|  | 13 | .792 |
|  | **17r** | **.474** |

The items in bold were removed based on their low primary loadings (λ<.50) or, in case of 12r, based on their substantial cross-loadings and residual covariances (cross-loading on Activity, expected parameter change = 251.82, residual covariance with item 13, EPC = 181.14). The new 10-item model showed the following fit: χ2 (32) = 367.850, *p* <.001, CFI = .958, RMSEA = .059, 90% RMSEA CI [.053, .064]).

| Table S2 |  |  |
| --- | --- | --- |
|  |  |  |
| *Item Loadings in the Modified 10-item EAS scale* | | |
|  |  |  |
| Factor | Item | Loading |
| Shyness | 1 | .605 |
|  | 8r | .784 |
|  | 14 | .783 |
|  | 20r | .619 |
| Emotionality |  |  |
|  | 11 | .558 |
|  | 15 | .848 |
|  | 19 | .681 |
| Activity |  |  |
|  | 4 | .628 |
|  | 9 | .488 |
|  | 13 | .818 |

| Table S3 |  | | | | | |  | | |
| --- | --- | --- | --- | --- | --- | --- | --- | --- | --- |
|  |  | | | | | |  | | |
| *Differences in Covariates Among Latent Classes* | | | | | | | | | |
|  |  | | | | | |  | | |
| Reference group | Average | | | | | | Active | | |
| Group | Active | | | Shy | | | Shy | | |
|  | OR | CI | *p* | OR | CI | *p* | OR | CI | *p* |
| Sex |  |  |  |  |  |  |  |  |  |
| Male | 1 (ref) |  |  |  |  |  |  |  |  |
| Female | 0.92 | [0.71, 0.83] | .347 | 1.09 | [0.80, 1.49] | .587 | 1.19 | [0.89, 1.59] | .241 |
| Family structure |  |  |  |  |  |  |  |  |  |
| Two-parent family | 1 (ref) |  |  |  |  |  |  |  |  |
| Single-parent family | 1.49 | [1.04, 2.15] | .031 | 1.10 | [0.57, 2.14] | .772 | 0.75 | [0.41, 1.35] | .334 |
| Education |  |  |  |  |  |  |  |  |  |
| Less than high school | 1 (ref) |  |  |  |  |  |  |  |  |
| High school/some college | 0.95 | [0.76, 1.19] | .666 | 1.58 | [1.02, 2.43] | .039 | 1.67 | [1.12, 2.49] | .013 |
| College degree | 0.65 | [0.49, 0.84] | .002 | 1.51 | [0.92, 2.47] | .104 | 2.33 | [1.46, 3.71] | <.001 |
| Maternal conflict | 1.06 | [0.95, 1.19] | .293 | 0.98 | [0.80, 1.20] | .852 | 0.92 | [0.76, 1.12] | .401 |
| Maternal attachment | 1.23 | [1.05, 1.43] | .009 | 1.02 | [0.10, 1.28] | .887 | 0.82 | [0.66, 1.02] | .072 |
